# Heat stress conditions affect the social network structure of free‐ranging sheep

**DOI:** 10.1002/ece3.10996

**Published:** 2024-02-13

**Authors:** Zachary Borthwick, Katrin Quiring, Simon C. Griffith, Stephan T. Leu

**Affiliations:** ^1^ School of Animal and Veterinary Sciences The University of Adelaide Roseworthy South Australia Australia; ^2^ School of Natural Sciences Macquarie University Sydney New South Wales Australia; ^3^ Department of Behavioural Ecology University of Göttingen Göttingen Germany; ^4^ School of Biological, Earth and Environmental Sciences University of New South Wales Sydney New South Wales Australia

**Keywords:** climate change, shade use, social behaviour, social networks, temperature humidity index, thermal environment

## Abstract

Extreme weather conditions, like heatwave events, are becoming more frequent with climate change. Animals often modify their behaviour to cope with environmental changes and extremes. During heat stress conditions, individuals change their spatial behaviour and increase the use of shaded areas to assist with thermoregulation. Here, we suggest that for social species, these behavioural changes and ambient conditions have the potential to influence an individual's position in its social network, and the social network structure as a whole. We investigated whether heat stress conditions (quantified through the temperature humidity index) and the resulting use of shaded areas, influence the social network structure and an individual's connectivity in it. We studied this in free‐ranging sheep in the arid zone of Australia, GPS‐tracking all 48 individuals in a flock. When heat stress conditions worsened, individuals spent more time in the shade and the network was more connected (higher density) and less structured (lower modularity). Furthermore, we then identified the behavioural change that drove the altered network structure and showed that an individual's shade use behaviour affected its social connectivity. Interestingly, individuals with intermediate shade use were most strongly connected (degree, strength, betweenness), indicating their importance for the connectivity of the social network during heat stress conditions. Heat stress conditions, which are predicted to increase in severity and frequency due to climate change, influence resource use within the ecological environment. Importantly, our study shows that these heat stress conditions also affect the animal's social environment through the changed social network structure. Ultimately, this could have further flow on effects for social foraging and individual health since social structure drives information and disease transmission.

## Graphical abstract

We GPS‐tracked 48 free‐ranging sheep in the arid zone of Australia. When heat stress conditions worsened, the social network was more connected (higher density) but with lower internal structure (lower modularity). Furthermore, an individual's social connectivity was affected by its shade use behaviour, and individuals with intermediate shade use were most strongly connected (degree, strength, betweenness).

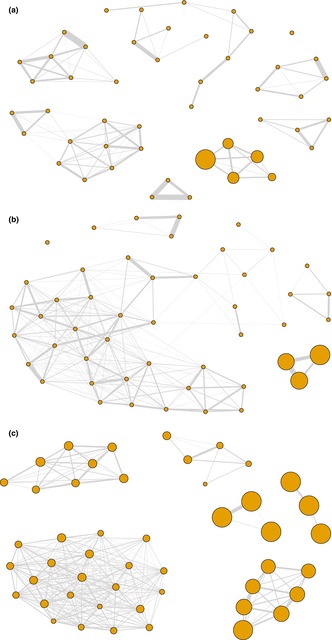

## 1 INTRODUCTION

The climate is changing, resulting in frequent and severe heatwaves, which will lead to conditions that could induce heat stress in many species (Sejian et al., 2018). Heat stress can have several effects on individual animals, including impaired endocrine and immune function (Lacetera, 2019; Mete et al., 2012) as well as reduced reproductive success (van Wettere et al., 2021). Heat stress also elicits changes in behaviour to avoid hyperthermia. Individuals can limit or change their activity patterns when ambient conditions are particularly challenging (Bourgoin et al., 2011; Fuller et al., 2016; Hetem et al., 2012; Leu et al., 2021). Similarly, animals can avoid microclimates that are hot and in direct sun and shift their space use to favour shaded areas (Hetem et al., 2012; Leu et al., 2021). Reduced food intake to decrease metabolic heat production, increased drinking frequency, and increased standing time to increase surface area for heat loss by convection (Galán et al., 2018) have also been recorded during hot conditions. Furthermore, how strongly individuals respond to changing ecological conditions can depend on individual attributes such as genetic and phenotypic traits, metabolic status (Galán et al., 2018), sex (Li et al., 2018), and body condition (Bright Ross et al., 2021).

Behavioural responses to heat stress reflect a cost–benefit trade‐off. For example, in Southern Pied Babblers (*Turdoides bicolor*) the trade‐off between foraging efficiency and heat‐dissipating behaviours has resulted in lower body mass (du Plessis et al., 2012). A similar trend was also seen in the Western Australian Magpie (*Cracticus tibicen dorsalis*) (Edwards et al., 2015), and alpine ibex (*Capra ibex*) (Mason et al., 2017). *Capra ibex* also display behavioural plasticity in space use and activity with females utilising altitudinal gradients and modifying their diel activity patterns to cope with heat stress conditions (Semenzato et al., 2021). These and other behavioural changes are considered the first response of individuals to heat stress (Sejian et al., 2018) and can increase the chances of survival (Li et al., 2018). However, thermal tolerance varies among individuals (Drown et al., 2021). Therefore, individuals that have social bonds, but different thermal tolerances will have to trade off optimal thermoregulation with their social propensity. This has the potential to impact group coordination and synchronised action which are important in social species affecting social network structure (Maldonado‐Chaparro et al., 2018). Social network structure in turn affects population processes such as social information (Aplin et al., 2012) and parasite transmission (Leu et al., 2020; Sah et al., 2017), as well as mating/reproductive success (Beck et al., 2021). Understanding how behavioural responses to heat stress conditions affect an individual's position in its social network and the social network structure of the group will provide important insight how climate change may affect population processes of social animal species. Here, we investigated how individual thermoregulatory behaviour in free‐ranging sheep in the arid zone of Australia affects individual social connectivity as well as the global social network structure of the study group.

During heat stress conditions, sheep spend time in the shade as a behavioural response to support physiological thermoregulation (Leu et al., 2021). Sheep also spend more time at watering points to avoid dehydration, and activity levels are substantially lower compared to more typical conditions (Leu et al., 2021). Sheep that were kept under tree shade showed fewer heat stress‐related behaviours and shade use behaviour reduced radiant heat load (De et al., 2020). Furthermore, individual sheep are repeatable in their shade use behaviour, and the length of time spent in the shade varies among individuals (Leu et al., 2021). We hypothesise that behavioural responses to heat stress conditions, in particular shade use, affect individual social association patterns, and the global social network structure of the study group. Furthermore, the individual response will likely be influenced by both its thermoregulatory requirements and its propensity to remain with its social group. How both are traded‐off remains to be investigated. Two alternative effects on the network are conceivable. Sheep could aggregate in a few large, shaded areas. This would result in social networks that are more connected (higher density) and less structured (lower network modularity). If individuals interact with conspecifics that are present in shade without social preference, this will lead to homogenised association patterns (low coefficient of variation of edge weights). Alternatively, if animals use several different shaded areas, networks are disrupted and become less connected. This would lead to lower network density, greater modularity and heterogeneous interaction patterns, that is, higher coefficient of variation of edge weights. We refrain from making a priori predictions as available shade was not limited but we do not know whether sheep would aggregate in a few or many separate shade patches. Both of these possible social outcomes will have very different consequences for the population as they will influence disease and information transmission very differently. We will discuss these outcomes in more detail further below.

## 2 MATERIALS AND METHODS

### 2.1 Study site and tracking of sheep

**FIGURE 1:**
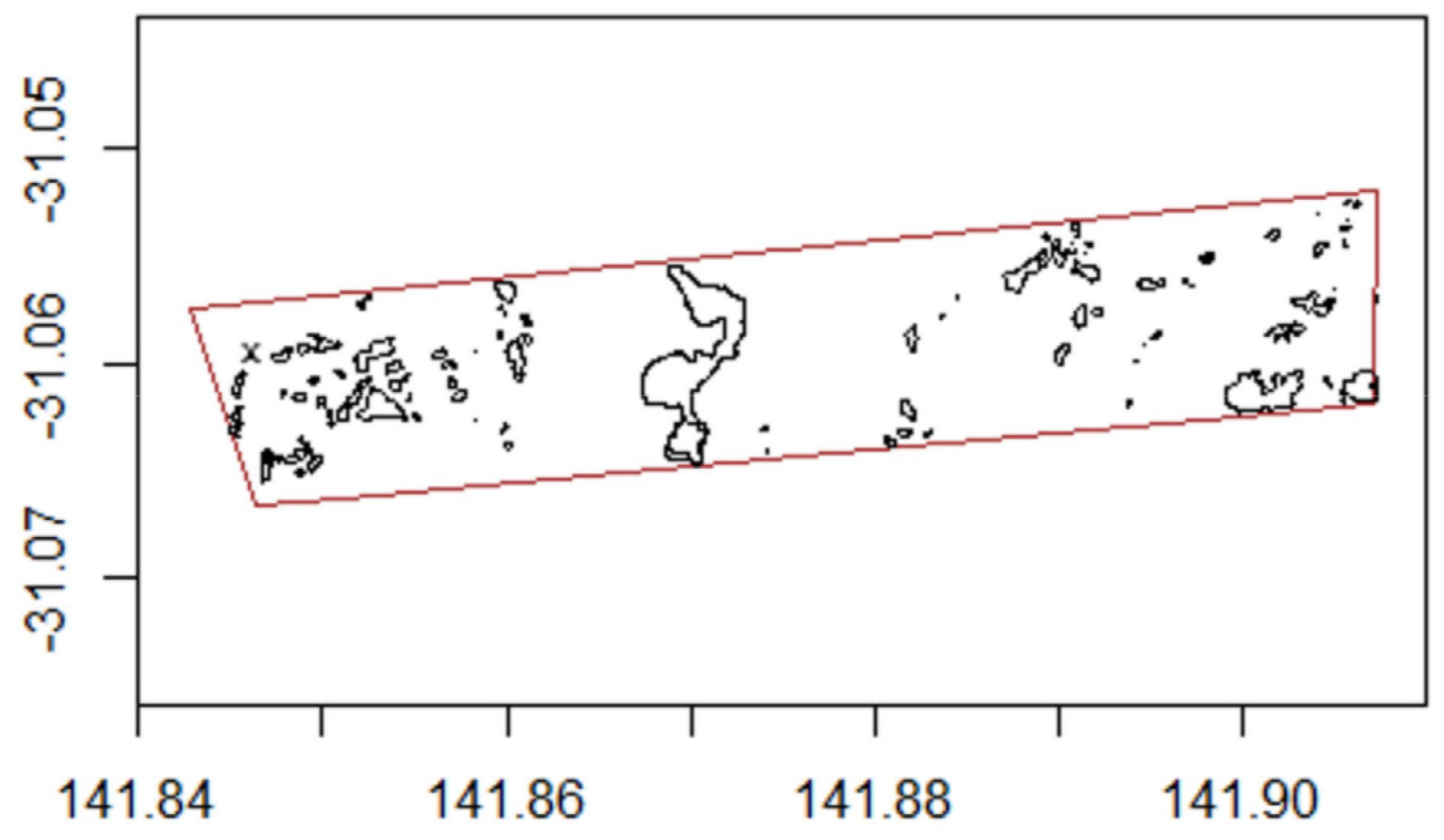
Map of the study paddock and its boundaries (red line). Shade patches are shown in black, the black x in the north‐western corner is the water point. The paddock measured 6 × 1 km in size, with longitude and latitude coordinates shown on the two axes. Reprinted from Leu et al., 2021, with permission from Elsevier.

We conducted this study at Fowler's Gap Arid Zone Research Station (31°05′ S, 141°43′ E). We observed 48 female sheep in a fenced paddock of 6 × 1 km for 82 consecutive days in summer 2018. The vegetation was chenopod shrubland, mostly consisting of blue bush (*Maireana* spp.) and small trees (*Acacia* spp.) that provided shade (Figure 1). The study area is part of the arid zone of Australia with a mean annual rainfall of 236 mm (year 1989–2018). The minimum and maximal temperatures during the 3‐h midday period ranged from 20.9 to 37°C, and 25.6 to 44.4°C respectively. All individual sheep were adult, sexually mature, non‐pregnant female Merinos, born in mid‐2016 and approximately 1.5 years old. They originated from the same flock and had the opportunity to be familiar with each other. Each individual was equipped with a GPS collar (Global Position System, i‐gotU GT‐120 by MobileAction, with a larger battery CE04381 by Core Electronics). The GPS collars recorded positions every 2 min. The GPS collars were programmed to record the location of all sheep synchronously. The collars weighed 700 g or 1.8% of the mean body mass of our sheep (39.3 ± SE 0.7 kg) and did not impact sheep movement behaviour (Leu et al., 2021). All sheep were treated using procedures approved by the University of New South Wales Animal Care and Ethics Committee in compliance with the Australian Code of Practice for the Use of Animals for Scientific Purposes (approval number 18/19B).

### 2.2 GPS data processing

We followed the same data processing procedure as in Leu et al. (2021). In brief, spatial outliers were removed from the raw GPS data using three methods, (1) all locations that used less than three satellites or were clearly outside the fenced study area; (2) locations that an individual could not have moved to at a maximal movement speed (180 m per 2 min i.e. 5.34 km/h; Manning et al., 2014) and (3) locations that resulted in spikes away from the movement path, filtered based on maximal speed and large turning angles. Because GPS data loggers recorded locations at slightly different times, we interpolated each individual's filtered data to exactly the same 2‐min intervals. After data processing, the dataset included a total of 352,075 GPS locations for all the individuals combined across the duration of the study. Individuals were recorded a mean of 7335 times (min 6733, max 7380). This represents a mean of 99.4% (min 91.2%, max 100%) of the theoretical possible number of 7380 locations, based on the observation length (246 h) and recording frequency (1 location per 2 min). We also tested the accuracy of the GPS units by placing 4 units into open habitat, which is dominating in the wider area. We recorded for 48 continuous hours. For each unit, we estimated its ‘true’ location by calculating the median latitude and longitude coordinates. We then calculated the positional error (accuracy) as the median distance between each fix and it the true location. The median accuracy was 1.67 m (Q1 = 1.11 m and Q3 = 2.55 m).

### 2.3 Inferring sheep social networks from spatial locations

We conducted all social network construction and statistical analysis using R statistical software (R Core Team, 2021). First, we considered two individuals to be associated if they were within one body length (approx. 1 m) of one another at the same time. Then, taking the accuracy (rounded to 2 m) of both GPS units into account, this leads to an association threshold of 5 m (body length 1 m + 2 × 2 m = 5 m). Using this threshold, we identified all associations among all possible pairs of individuals and constructed a social network for the midday period of each study day (*n* = 82). We focussed on a 3‐h midday period with 1.5 h before and after the zenith of the sun, as radiation is highest and shade use would be the most beneficial at this time (Leu et al., 2021). Hence, each sheep was observed for a total of 246 h across the study period; 3 h each day for 82 consecutive days.

We used the R package spatsoc (Robitaille et al., 2019) to apply the spatio‐temporal criteria and generated a group by individual matrix (GBI) for all dyads. Then, using the package asnipe (Farine, 2013), we generated an individual by individual adjacency matrix and calculated the simple ratio association index as edge weight. The edge weight reflects the association strength between the two individuals of a dyad, it is calculated as the proportion of instances a dyad spent in close contact relative to the total number of instances they were both recorded, this includes by themselves, associating with others as well as each other (Hoppitt & Farine, 2018).

For each midday network, we calculated three whole network measures—network density, modularity and coefficient of variation of edge weights. We used the package igraph (Csárdi & Nepusz, 2006) to calculate the network density for each midday network. Network density is the ratio between the number of existing edges connecting individuals and the number of all theoretically possible edges in this network. The value ranges from 0 to 1 and a high value of network density indicates a well‐connected network (Sosa et al., 2021). Second, we calculated modularity as it gives insight into the internal structure of the social network and measures the extent to which a network is divided into different groups or clusters (Sosa et al., 2021). Networks with high modularity are characterised by many and strong connections within the group or cluster, while connections between groups are sparser (Tetzlaff et al., 2023). Fission–fusion societies, such as sheep (Della Libera et al., 2023), are structured into sub‐groups and modularity measures are higher compared to cohesive groups (Sosa et al., 2021). If divergent shade use results in greater internal network structure, and stronger subgroup clustering, we would expect higher modularity measures. We calculated modularity using the igraph package with the algorithm specified in Clauset et al. (2004). Third, we calculated the coefficient of variation of edge weights. The coefficient indicates how heterogeneous dyadic relationships were across the social network. A greater coefficient of variance indicates greater heterogeneity, that is some pairs were together on more instances than other pairs. This can either be consecutively during few but long periods, or during many short periods. This metric is particularly informative alongside density and modularity. Density and modularity inform about the structure of the whole network, while the coefficient of variance of edge weights informs how structured individual associations are. We then related these measures to the temperature humidity index (THI) (see below) which is a proxy for the ambient heat conditions. We have previously shown that network density can be influenced by environmental characteristics such as habitat structural complexity (Leu et al., 2016). Here, we were interested in whether shaded areas and their use influence the network.

In addition to the whole network level, we also investigated the effect of heat stress conditions at the individual level. For each individual, in each 3‐h midday network, we calculated three node‐level metrics—degree, strength, and betweenness centrality. We related these measures to individual shade use behaviour. Degree represents the number of individuals an animal associates with during the given time period, strength is the sum of edge weights of the node (Sosa et al., 2021). Betweenness centrality measures the number of times a node is included in the shortest paths between two other nodes (Sosa et al., 2021). Hence, it reflects the extent to which an individual connects sub‐groups. It indicates the relative importance of an individual for transmission processes, for example, transmission of information or pathogens (Newman, 2005).

### 2.4 Calculating THI and individual shade use

Different bioclimatic indices exist to assess the impact of climatic conditions on livestock reared outdoors (Theusme et al., 2021). In this study, we used the THI which is a suitable climatic parameter to describe heat stress conditions in sheep (Marai et al., 2007; Ozella et al., 2020; Theusme et al., 2021). It is calculated as THI _sheep_ = T – {0.31 – (0.31 × (RH/100)) × (T – 14.4)}; where T = Ambient Temperature (in °C) and RH = Relative Humidity (in %). The dry bulb temperature and relative humidity were measured every hour at a nearby Bureau of Meteorology Automated Weather Station (Fowlers Gap field station AWS41628). The weather station and our study site were 17.2 km apart and we have previously shown that the recorded weather data were representative for our study site (Leu et al., 2021). Using the weather station data, we calculated the mean THI for the 3‐h midday period around the zenith of each day. In our analysis, we used THI as a continuous variable. Nevertheless, different THI thresholds have been identified to indicate different levels of heat stress for sheep; no heat stress <22.2 THI, moderate heat stress 22.2 to <23.3, severe heat stress 23.3 to <25.6 and extreme severe heat stress ≥25.6 (Marai et al., 2007).

We also calculated how long each individual sheep spent in the shade during the 3‐h midday period of each day. We considered that trees with a minimum height of 1 metre provide shade for a standing sheep. We mapped 106 tree patches using a handheld GPS unit (Leu et al., 2021). Each tree patch was a georeferenced polygon with known boundary coordinates (Figure 1). For each sheep, we determined the number of GPS locations that were inside any of the 106 tree patches. Reflecting the consistent sampling interval (1 location every 2 min), one location represents on average approximately 2 min spent inside the shaded polygon. For each 3‐h midday period, we determined the total number of GPS locations inside any polygon and multiplied it by 2 as a proxy for the total time spent in the shade (in minutes).

### 2.5 THI and shade use effects on network structure

We used three linear models to test the effect of THI (continuous variable) on whole network metrics (*density, modularity and coefficient of variation of edge weights*—all continuous variables). We then determined how THI (continuous) affects individual shade use behaviour (minutes in shade per 3‐h midday period, continuous) using a linear mixed model. Time spent in shade was the dependent variable, THI the independent variable and sheep *ID* (48 levels) a random effect. Finally, we investigated whether shade use behaviour affected an individual's social network connectedness. We scaled and centred the shade use variable using the ‘scale’ function in base R (R Core Team, 2021) to meet model assumptions. We used three linear mixed models. *The models with strength* and *betweenness centrality* (both continuous) as the dependent variable used a Gaussian distribution, whereas the model for *degree* (discrete count data) used a Poisson distribution. We included *shade use* and (*shade use*)^2^ both as fixed effects (continuous) as this better followed the data structure and improved model fit. As before, sheep *ID* (48 levels) was a random effect.

### 2.6 Permutation‐based hypothesis testing

Social network measures are inherently non‐independent and therefore violate an important assumption of most statistical tests (Croft et al., 2011). Combining linear models with permutations accounts for the non‐independence and also allows to identify the effect of the investigated variable beyond other (unmeasured) variables that contribute to the social structure (Farine, 2017). We created 1000 random social networks for each analysis, deduced the same network measures and reran the same linear models. We then compared the model coefficient based on the empirical social network with the distribution of coefficients derived from the random networks. Two‐sided‐*p*‐values for each effect were calculated as twice the number of times a randomised coefficient was more extreme than the observed test statistic divided by 1000, the number of randomisations (Farine, 2017).

We conducted pre‐network permutations when investigating whole network metrics. We used the ‘network_permutation’ function in asnipe (Farine, 2013). This involved carrying out 1000 randomisations of the group‐by‐individual (GBI) matrices, which assigned individuals to different, random dyads. Using these randomised GBI matrices, we generated 1000 randomised adjacency matrices and deduced the relevant network measures. We conducted node label randomisations when investigating node‐level metrics. We used the ‘perm.net.nl’ function in the ANTs package (Sebastian et al., 2018). We used shade use as node labels and directly randomised the shade use values to generate 1000 shuffled data sets for the midday period of each day.

## 3 RESULTS

### 3.1 Heat stress conditions and shade use

Across the 82 days of the study period, individuals experienced on average a THI of 28.9 (SD = 3.5) with a min of 20.72 and max of 34.29. Most days (81.71%) fell into the extreme severe heat stress category, followed by 10.98% in severe heat stress, 4.88% in moderate heat stress and 2.44% in the no heat stress category. The average tree height was 2.23 metre and 84 of the 106 shaded areas were used by our sheep. Individual shade use behaviour increased with THI (estimate = 6.88, SE = 0.26, *t*‐value = 26.61, *R* ^2^ = .16, *p* < .001). During the midday period (180 min) sheep spent a mean of 66.9 min in the shade when conditions were categorised as extreme severe heat stress compared to only 20.8 min when conditions were classified as no heat stress.

### 3.2 Heat stress conditions and shade use affect network structure

**FIGURE 2:**
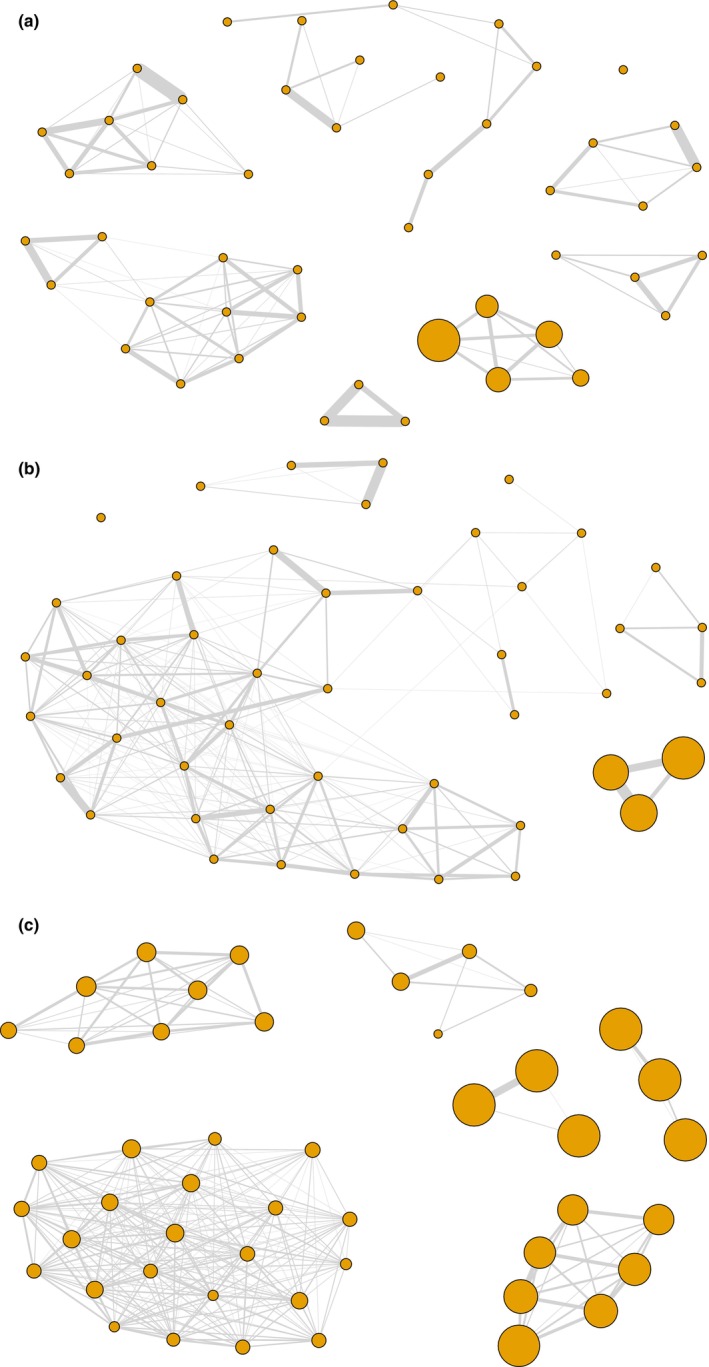
Social networks at different temperature humidity indices, (a) no heat stress for sheep (THI 21.85), (b) lower end of severe heat stress (THI 23.34), (c) extreme severe heat stress (THI 30.75). Individual sheep are represented as nodes, with the node size scaled by shade use. Larger node size represents more time spent in the shade, during the 3‐h midday period. In (a) shade use ranged from 0% (smallest node size) to 18.9% (largest node size) of the 3 h, 0% to 81.1% in (b), and 2.2% to 100% in (c). Edge weights represent the association strength between two individuals. Thicker edges indicate a stronger association, measured as single ratio association index (SRI, proportion of incidents recorded together). Individual connectedness (degree) is highest for sheep with average shade use, while it is lower for individuals with below or above average shade use.

**FIGURE 3:**
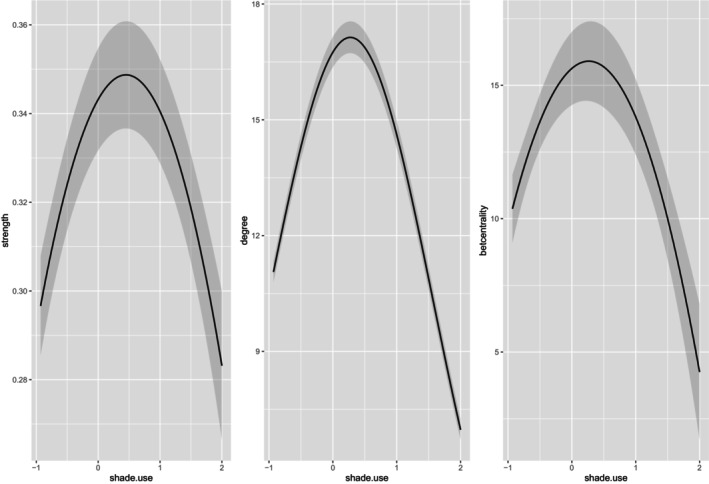
Estimated values of node strength, degree and betweenness centrality against shade use (scaled and centred) are depicted in three separate graphs.

Network density increased with THI, whereas modularity and coefficient of variation of edge weights decreased (Table 1). Figure 2 illustrates networks at different THIs, ranging from no heat stress to extreme severe heat stress for sheep. Shade use was associated with all three node‐level social network measures. More specifically, the effect of shade use and (shade use)^2^ was comparable for degree, strength and betweenness centrality (Table 2). The combination of the positive coefficient for shade use and negative coefficient for (shade use)^2^ means that as shade use increased, sheep showed greater social connectedness, which then decreased again when individuals spent even longer periods in the shade (Figure 3).

**TABLE 1:** Effects of temperature humidity index (THI) on whole network structure.

Dependent variable	Coefficient	SE	*t*	*R* ^2^	*P*_rand_
Network density	0.02	0.005	3.82	.14	<0.001
Modularity	−0.014	0.005	−2.699	.07	<0.001
CV of edge weights	−0.14	0.03	−5.09	.24	<0.001

**TABLE 2:** Effect of shade use and (shade use)^2^ on node‐level network metrics.

	Degree				Strength				Betweenness centrality			
	Coeff	SE	*Z*	*P*_rand_	Coeff	SE	*t*	*P*_rand_	Coeff	SE	*t*	*P*_rand_
Shade use	0.17	0.006	27.38	<0.001	0.025	0.003	7.49	<0.001	2.05	0.58	3.54	0.006
(Shade use)^2^	−0.30	0.006	−49.46	<0.001	−0.027	0.003	−8.70	<0.001	−3.87	0.55	−6.99	<0.001

## 4 DISCUSSION

### 4.1 Heat stress conditions affect whole network structure

As heat stress conditions worsened, indicated by an increasing THI, networks became denser, less modular and the animals' social associations became less heterogeneous (decrease in CV of edge weights). That is, the differentiation into stronger and weaker social ties was reduced and social associations were more similar. Taken together, this suggests that with increasing heat stress conditions, the social network was more connected but lost internal structure. This is consistent with an earlier study that found a negative, but non‐significant, relationship between THI and clustering coefficient (a different measure for internal structure). One potential explanation for the lack of significance could be the lower THI measures reflective of the study site in central Europe (Ozella et al., 2020). External stressors can impact social network structure. For instance, Maldonado‐Chaparro et al. (2018) showed that even temporary disruptions, such as splitting a group of zebra finches into sub‐groups for 2 days affected the social network after the sub‐groups were joined again. However, in contrast to our study, the disturbance effect of group splits increased the internal group structure and caused more differentiated social relationships. This further reduced foraging efficiency as fewer individuals accessed the foraging patches at the same time (Maldonado‐Chaparro et al., 2018). The more connected and less structured network in our study could lead to improved transmission processes (Sah et al., 2017) which can be beneficial or costly depending on the transmitted agent. Improved information transmission can increase the capacity of individuals to find resources such as food or water (Aplin et al., 2012; Kendal et al., 2015) and in our study, shade. Furthermore, predator detection could also be improved (Frechette et al., 2014). For example, although aggregations are more vulnerable to being detected by predators (Pulliam, 1973), faster information spread about the presence of predators could provide protection from actual predation. Furthermore, in the context of this study, shaded areas are likely low‐risk as they provide cover, which reduces detection by predators and reduces glare that could hamper the prey's ability to monitor its environment (Carr & Lima, 2014; Sabal et al., 2021).

Network density and modularity have been shown to be negatively related (Puga‐Gonzalez & Sueur, 2017). In our study, this could suggest that THI does not independently affect these measures. Regardless, a well‐connected social network with low modularity also improves the spread of pathogens and parasites, which has negative well‐being and health consequences (Griffin & Nunn, 2012; Nunn et al., 2015). Furthermore, disease transmission may be particularly detrimental when animals are already heat‐stressed, as stress has been shown to impair immune system function (Hing et al., 2016). Hence, the costs of a connected and homogeneous network structure could outweigh the benefits, even if heat stress conditions are rare and short‐lived. This is because pathogen transmission processes often only require a few short interactions. However, the cost–benefit ratio of highly connected networks in sheep remains to be investigated.

### 4.2 Shade use behaviour affects individual network connectivity

Sheep varied in the extent of their shade use behaviour. During periods of extreme severe heat, time spent in the shade ranged from 0 to 180 min over the 3‐h period. We previously showed that shade use behaviour varies among individuals and that individuals are consistent in their own behaviour between heat wave events (Leu et al., 2021). Competition among individuals, which can be further modulated by the social hierarchy, can influence access to resources (Majolo et al., 2012; Nelson‐Flower et al., 2011). However, this is not the case in all species. In the sociable weaver (*Philetairus socius*) for instance, which lives in complex cooperative societies, shade use behaviour, and hence access to shade, was not influenced by social rank (Cunningham et al., 2017). Nevertheless, dominant social weaver individuals could maintain their body temperature more precisely at increasing ambient temperatures, whereas subordinate individual could not (Cunningham et al., 2017). At this stage, we do not know whether social hierarchy plays a role in the shade use behaviour in sheep. For example, whether high ranking individuals restrict access to certain patches of shade. This was beyond the scope of the current study. Instead, we were interested in how the variation in using shade patches affects social connectivity in sheep.

When ambient conditions (temperature, THI) are more challenging, sheep spend more time with conspecifics (Doyle et al., 2016) and social networks are more connected (as seen in this study). Furthermore, we identified that shade use, as a behavioural response to heat stress conditions drives change in social behaviour and connectivity. Interestingly, shade use had a non‐linear relationship with an individual's social connectivity. First, social connectivity increased with shade use behaviour, before reaching a peak and then decreasing again. This was the case for node degree, strength and betweenness. The initial increase in social connectedness may reflect fusion events with some sub‐groups aggregating at certain shade patches. The subsequent decrease in node degree could be attributed to fission events with individuals with low preference for shade seeking behaviours wandering away from the shade. Our paddock included 106 tree patches, 84 of those patches were used during the study period. This suggests that shaded areas were unlikely limited and that the observed behavioural changes were not driven by limited habitat options. Two different mechanisms could then underlie the choice for certain shade patches. Either sheep make active social choices due to who else is present a given shade patch. Alternatively, sheep choose patches based on their different environmental qualities which would result in indiscriminate social associations during heat stress conditions. Our results of reduced internal network structure (lower modularity and CV of edge weights) with increasing THI may support the latter notion. More research would be needed to differentiate between the two mechanisms.

Sheep with intermediate shade use behaviour were most strongly connected in the social network. Whereas individuals that spent either less or more time in the shade were less connected. One possible explanation is, that those intermediate individuals spent some time in one shade patch, then moved away into the open where they may encounter and interact with individuals that remained active and spent little time in the shade. This is then followed by another period in the shade, either in the same or a different shade patch. Furthermore, sheep with intermediate shade use behaviour appear to play an important role in the overall social network connectivity. This notion is supported by our finding that intermediate shade users also had the highest betweenness values. Highly connected individuals (high degree and strength) in important network positions (high betweenness) play a crucial role for maintaining network cohesion, stability and for social transmission processes (Wey et al., 2008). For instance, in pigtailed macaques (*Macaca nemestrina*), a small subset of well‐connected individuals significantly contributes to maintaining stable social environments during periods of chronic perturbations. Without these individuals, group members build smaller, less diverse, and less integrated networks (Flack et al., 2006). In such situations where some individuals are responsible for maintaining links between groups that are otherwise less connected, weak ties emerge which can serve as transmission points (Vanderwaal et al., 2016). As mentioned above this could be beneficial for the population if information is transmitted or costly if parasites or pathogens are transmitted. Irrespective of the agent that is transmitted, it shows the disproportionate influence of these individuals for population processes.

This study raises important practical considerations for animal welfare and management in the face of heat stress. While this study explored intra‐population variability in responses to heat stress, future studies could investigate the effect of varying levels of shading between paddocks and populations. In conclusion, heat stress conditions affected shade use behaviour in sheep, their individual social connectedness and group social network structure. Importantly, individuals with intermediate shade use behaviour occupied well‐connected and important network positions. This suggests that they play an important role in maintaining social network cohesion and function during periods of heat stress. Our study shows that climate change which is predicted to lead to more frequent and more severe heat stress conditions, may not only impact the ecological environment that individuals experience but also their social environment through changes in the social network structure.

## AUTHOR CONTRIBUTIONS

**Zachary Borthwick:** Conceptualization (equal); data curation (equal); formal analysis (equal); writing – original draft (equal). **Katrin Quiring:** Data curation (equal); formal analysis (equal); writing – review and editing (equal). **Simon C. Griffith:** Conceptualization (equal); writing – review and editing (equal). **Stephan T. Leu:** Conceptualization (equal); data curation (equal); formal analysis (equal); funding acquisition (lead); writing – review and editing (equal).

## CONFLICT OF INTEREST STATEMENT

The authors have no conflict of interest to declare.

## Data Availability

The data supporting the findings of this study are availbale on Dryad, https://doi.org/10.5061/dryad.qfttdz0qp.
